# Hormonal Changes after R-CHOP Chemotherapy in Patients with Diffuse Large B-Cell Lymphoma: A Prospective Study

**DOI:** 10.3390/medicina58060710

**Published:** 2022-05-26

**Authors:** Worapaka Manosroi, Jiraporn Chirayucharoensak, Chatree Chai-adisaksopha, Phichayut Phinyo

**Affiliations:** 1 Division of Endocrinology, Department of Internal Medicine, Faculty of Medicine, Chiang Mai University, Chiang Mai 50200, Thailand; c.jiraporn.pui@gmail.com; 2 Department of Internal Medicine, Pichit Hospital, Pichit 66000, Thailand; 3 Division of Hematology, Department of Internal Medicine, Faculty of Medicine, Chiang Mai University, Chiang Mai 50200, Thailand; oddmed@hotmail.com; 4 Clinical Epidemiology and Clinical Statistic Center, Faculty of Medicine, Chiang Mai University, Chiang Mai 50200, Thailand; phichayutphinyo@gmail.com; 5 Department of Family Medicine, Faculty of Medicine, Chiang Mai University, Chiang Mai 50200, Thailand

**Keywords:** diffuse large B-cell lymphoma, hormonal changes, chemotherapy

## Abstract

*Background and Objectives*: There is a lack of data regarding hormonal metabolic abnormalities resulting from the R-CHOP regimen (rituximab, cyclophosphamide, doxorubicin, vincristine, prednisolone), a commonly used chemotherapy in diffuse large B-cell lymphoma (DLBCL). This study aimed to determine the incidence of hormonal and metabolic changes after R-CHOP therapy. *Methods and Methods*: This prospective cohort study initially included 17 DLBCL patients. Hormonal tests, including gonadal function, thyroid function, and ACTH stimulation tests for cortisol and metabolic markers, were performed before the 1st and after the 5th cycle of R-CHOP. The paired *t*-test was used to evaluate the changes. Statistical significance was set at *p* < 0.05. *Results*: Out of 17 patients, two died before the last follow-up, and 15 completed the study. LH levels were significantly higher in males after the 5th cycle (*p* = 0.04), while no significant changes were observed in other hormonal levels. After the 5th cycle, the only trend toward statistical significance was observed with higher FSH in males, higher blood glucose, and cholesterol. After the 5th cycle of chemotherapy, seven patients had at least one hormonal dysfunction, three patients had alterations in their thyroid function tests. Three patients had adrenal insufficiency (AI), one of whom also had secondary hypothyroidism concomitant with hypogonadism and AI. Additionally, two males had hypogonadism, and impaired fasting glucose was observed in three patients. *Conclusions*: Hormonal and metabolic abnormalities can occur in DLBCL after the 5th R-CHOP cycle. A high level of awareness and careful observation is of value in detecting these abnormalities, as some can be lethal.

## 1. Introduction

Diffuse large B-cell lymphoma (DLBCL) is one of the most common lymphoma subtypes. An R-CHOP regimen (rituximab, cyclophosphamide, doxorubicin, vincristine, prednisolone) is the mainstay therapy for DLBCL [1]. This regimen is administered once every 21 days for an average of six cycles. This combination therapy can lead to long-term disease control in approximately 90% of early-stage disease cases and 60% of advanced-stage disease cases [1].

Commonly reported side effects of R-CHOP include alopecia, nausea, fatigue, and changes or reduction in the sense of taste [2]. Additionally, each drug in the R-CHOP regimen has its own set of potential adverse effects. Rituximab has been reported to mediate the reduction in TSH receptor antibodies and increase the remission rate of Graves’ disease [3]. It has also been reported to help delay the deterioration of ß-cell function in type 1 diabetes [4]. Cyclophosphamide can lead to gonadal suppression, which can occur in both men and women after using high-dose cyclophosphamide [5]. Transient gonadal dysfunction (oligospermia and azoospermia) has been reported following doxorubicin therapy [6]. The syndromes of inappropriate antidiuretic hormone secretion (SIADH) and transient infertility have been reported to be related to vincristine therapy [6]. Multiple hormonal dysfunctions have been documented with long-term and high-dose use of prednisolone. The most common effect on the hormonal axis is suppression of the hypothalamic–pituitary–adrenal (HPA) axis, which can lead to adrenal insufficiency (AI) [7]. Alteration of thyroid hormones, including the transient suppression of free T3 (FT3), free T4 (FT4), and TSH, have also been reported after the use of glucocorticoids [8]. Additionally, decreased production of gonadotropin-releasing hormone (GnRH) by the hypothalamus resulting in reduced production levels of LH, FSH, estradiol, and testosterone have been demonstrated following the administration of glucocorticoids [9].

In one prospective study, ten patients with DLBCL who had received 6–8 cycles of the R-CHOP/CHOP regimen had an AI incidence rate of about 30% after completing a course of this regimen. The incidence was reported to be highest after the 5th cycle of chemotherapy [10]. Furthermore, several symptoms of hormonal abnormalities following chemotherapy have been under-recognized. Consequently, these abnormalities may lead to life-threatening conditions. Data on other hormonal alterations after an R-CHOP regimen, including glycemic and metabolic abnormalities, are still lacking. This study aimed to evaluate the changes in hormones, basic metabolic panel results, and nutritional status and determine the incidence of hormonal dysfunctions after the 5th cycle of the R-CHOP regimen in newly diagnosed DLBCL patients.

## 2. Materials and Methods

This prospective cohort study was conducted in a tertiary care medical center between March 2021 and February 2022. The study was approved by the local ethical committee of the Faculty of Medicine, Chiang Mai University (Ethical number: MED-2563–27808, Date: 15 February 2021). Informed consent was obtained from all participants included in the study. All procedures were conducted in accordance with the ethical standards of the responsible committees on human experimentation (institutional and national) and with the Declaration of Helsinki 1975 as revised in 2008.

All 17 participants were recruited from the outpatient hematology clinic. The inclusion criteria were: (1) newly diagnosed DLBCL patients aged > 18 years and (2) scheduled to receive the R-CHOP chemotherapy regimen. The exclusion criteria were patients with (1) a history of using glucocorticoids or any medical compound containing glucocorticoids within one month prior to the recruiting period; (2) known pituitary diseases, including a history of pituitary radiation therapy or surgery; (3) AI, adrenal mass or adrenal metastasis; (4) known thyroid disease, e.g., thyrotoxicosis, hypothyroidism, or ongoing treatment of thyroid diseases; (5) a history of ovarian or testicular diseases or previous ovarian or testicular surgery; (6) currently using any medicinal form of contraception; and (7) currently using any medication that interferes with hormonal measurement.

**Figure 1:**
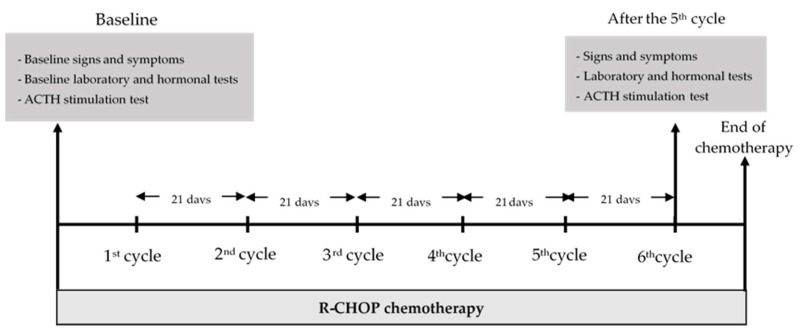
Diagram of the study.

Demographic data together with the staging of DLBCL, ECOG performance status [11], signs and symptoms of hormonal changes, nutritional status assessed by Nutrition Alert Form (NAF) [12], percentage of body water, muscle mass, and visceral fat level were obtained before the 1st cycle and at day 21 after the 5th cycle of R-CHOP, which was the day before the start of the 6th cycle of R-CHOP. Laboratory investigations, biochemical data, and hormonal investigations, including the ACTH stimulation test for serum cortisol, FT3, FT4, TSH, FSH, LH, estradiol (for females), and testosterone (for males), were evaluated before the 1st cycle and at day 21 after the 5th cycle of R-CHOP therapy. A diagram of the study is shown in Figure 1. The cumulative dose of prednisolone used in each cycle of R-CHOP was 100 mg per cycle.

### 2.1. Laboratory Assays and ACTH Stimulation Tests

Serum FT3 (normal reference range, 2.04–4.40 pg/mL), FT4 (normal reference range, 0.93–1.71 ng/dL), and TSH (normal reference range, 0.27–4.2 μIU/mL) were measured by electrochemiluminescence assay (ECLIA) (Elecsys^®^ FT3 III, FT4 III, TSH assay, Roche Diagnostics GmbH, Mannheim, Germany) with intra- and inter-assay variation of 1.9–8.2%. Serum cortisol levels were measured by electrochemiluminescence assay (ECLIA) (Elecsys^®^ Cortisol II assay, Roche Diagnostics GmbH, Mannheim, Germany). The intra- and inter-assay coefficients of variation for serum cortisol were <10%. FSH (normal reference range, 1.5–12.4 IU/L in men, 3.5–12.5 IU/L in follicular phase women, 1.7–7.7 IU/L in luteal phase woman, 25.8–134.8 IU/L in menopausal women), LH (normal reference range, 1.7–8.6 IU/L in men, 2.4–12.6 IU/L in follicular phase women, 1.0–11.4 IU/L in luteal phase women, and 7.7–58.5 IU/L in menopausal women) were obtained using an electrochemiluminescence assay (ECLIA) (Elecsys^®^ LH, FSH assay, Roche Diagnostics GmbH, Mannheim, Germany method with an intra- and inter-assay of variation of 1.6–5.2%. The serum testosterone test (normal reference range, 0.93–7.4 ng/mL) was performed using the electrochemiluminescence assay method (ECLIA) (Elecsys^®^ Testosterone III assay, Roche Diagnostics GmbH, Mannheim, Germany) with an intra- and inter-assay of variation of 1.3–11.5%. Serum estradiol (normal reference range, 12.4–233 pg/mL in follicular phase women, 22.3–341 pg/mL in luteal phase women, <5.0–138 pg/mL in menopausal women), was performed using the electrochemiluminescence assay method (ECLIA) (Elecsys^®^ Estradiol III assay, Roche Diagnostics GmbH, Mannheim, Germany) with an intra- and inter-assay of variation of 1.8–12.3%. Muscle mass, body water, and visceral fat level were measured using the Xiaomi MI scale 2 (Bioelectrical impedance analysis technology, Mi Ecosystem company, Anhui, China). The correlation coefficients with X-SCAN PLUS 970 were 0.969, 0.945, and 0.949, respectively.

The low-dose ACTH stimulation test was performed to determine adrenal function status. Serum cortisol concentrations were measured before, and at 20 and 40 min after 5 µg of ACTH was administered intravenously. The 5 µg of ACTH was prepared by hospital pharmacists by diluting a 250 µg ampule of ACTH in normal saline and storing the mixture at 2–8 °C. Solutions were used within 60 days of preparation.

### 2.2. Definitions

According to the newest generation of cortisol assay, AI was defined as a peak serum cortisol level after a low-dose ACTH stimulation test of <14.5 µg/dL [13]. Secondary hypothyroidism was defined as FT4 and FT3 lower than their respective normal ranges together with either a suppressed TSH level (<0.27 μIU/mL) or a normal TSH level. Non-thyroidal illness (NTI) was defined as FT3 lower than the normal range with no alteration of TSH level. Suppressed TSH level or subclinical hyperthyroidism was defined as an isolated low TSH level with no alteration of FT3 or FT4 levels. Subclinical hypothyroid was defined as an isolated higher than the normal range (>4.2 μIU/mL) of TSH level without alteration of FT3 or FT4. Significant weight loss was defined as weight loss > 10% in 6 months. Hypogonadism was defined by low FSH, LH, testosterone (<3 ng/mL in males), estradiol (<40 pg/mL in females) plus the signs and symptoms of hypogonadism, e.g., amenorrhea for more than 3 cycles in reproductive females, and loss of libido, erectile dysfunction and/or loss of morning erection in males. Menopause was defined as women who had no menstrual periods for at least 12 months prior to the study or who had had their uterus removed. AI symptoms were defined as nausea/vomiting, lethargy, and orthostatic hypotension. Hypothyroidism symptoms were defined as having at least 3 of the following symptoms: constipation, cramping, excessive weight gain, edema, depression, or slow thought. Hyperthyroidism symptoms were defined as having at least 3 of the following symptoms: palpiations, tremors, excessive sweating, unexplained weight loss, and irritability. Hyperglycemia with diabetes-range glucose was diagnosed based on fasting blood glucose > 126 mg/dL. Impaired fasting glucose was defined as fasting blood glucose ≥100 mg/dL. Hyponatremia was defined as serum sodium < 135 mEq/L. Malnutrition was diagnosed by an NAF score of more than 5 [12]. B symptoms were any report of fever, night sweats, or weight loss. Bulky disease was defined as DLBCL with a tumor size ≥7.5 cm. ECOG performance status was graded from 0 to 5 [11]. The staging of DLLBCL was categorized by Ann Arbor staging [14].

### 2.3. Statistical Analysis

The data were analyzed by STATA version 17.0 (StataCorp, College Station, TX, USA). The statistically significant level was set at *p*-values < 0.05. For categorical variables, counts or percentages are reported; for normally distributed continuous variables, means and standard deviation (SD) are presented. For non-normally distributed continuous variables, medians with interquartile ranges are reported. The paired *t*-test was used to identify changes in normally distributed continuous data, while the Wilcoxon signed-rank test was used for non-normally distributed continuous data before and after the chemotherapy regimen. The sample size was calculated based on a previous report by Owattanapanich et al. using the changes in levels of serum cortisol between AI and non-AI groups and a ratio of AI to non-AI of 1:7 [10]. The power analysis option for a two-sample comparison of means with repeated measures was used to determine the required sample size. A sample size of at least 2 patients with AI and 13 with non-AI was estimated to give 90% power at the 5% significance level (one-sided).

## 3. Results

### 3.1. Baseline Characteristics

Seventeen patients with newly diagnosed DLBCL were enrolled in this study. Of these, two patients unexpectedly died before receiving the 5th cycle of chemotherapy. Therefore, 15 patients (six males and nine females) completed the study. The median age was 60.6 ± 12.4 years (range 34 to 80 years). The mean BMI was 21.7 ± 4.4 kg/m^2^. Three patients had fairly low blood pressure, with systolic pressure < 100 mmHg; among these, one had orthostatic hypotension. Eight patients had comorbidities, including diabetic mellites (26.7%), hypertension (40.0%), dyslipidemia (26.7%), chronic kidney disease (6.7%), and chronic hepatitis C infection (6.7%). Most patients (66.7%) had a good ECOG performance status of 0–1 points. According to Ann Arbor staging, nearly two-thirds of the patients (66.7%) were in stage 3 or 4. B-symptoms were reported in three patients (20.0%) prior to receiving the regimen. More than 80% of the patients had bulky disease. Two patients reported at least one symptom of AI. Among the female patients, eight were in the menopausal stage, and one was in the reproductive phase with no reported secondary amenorrhea prior to chemotherapy. None of the males reported hypogonadal symptoms before chemotherapy. No patients had symptoms of hypoglycemia or unexplained significant weight loss prior to the 1st dose of chemotherapy.

One patient had subclinical hyperthyroidism (No. 5), and two had NTI (Nos. 10 and 14), while the other 12 patients were euthyroid before chemotherapy. Among the females, eight had LH, FSH, and estradiol corresponding with their menopausal stage. The reproductive female showed normal gonadal function (No. 2). One male had low testosterone levels (No. 10) and low LH and FSH, indicating secondary hypogonadism from andropause. In adrenal function, no AI was documented in any of the patients before chemotherapy. Hyponatremia was documented in one patient and hypokalemia in one patient before chemotherapy.

As for the metabolic parameters, among patients without prior diabetes mellitus, 4 out of 11 had blood glucose in the impaired fasting glucose range (Nos. 4, 9,12, and 14). Of the patients with diabetes, fasting blood glucose levels ranged from 86–136 mg/dL. Regarding nutritional status, two patients had a moderate degree of malnutrition (Nos.1 and 11).

A summary of patient characteristics and all laboratory values are shown in Table 1, Table 2 and Table 3 and Supplementary Table S1.

### 3.2. Hormonal Changes and Hormonal Dysfunctions before the 1st and after the 5th R-CHOP Chemotherapy

Among 15 patients, 7 patients had at least one hormonal dysfunction after the 5th R-CHOP therapy. There was no significant difference in TSH, FT3, or FT4 levels before the 1st or after the 5th cycle of chemotherapy. Among patients with euthyroid before the 1st cycle of chemotherapy, two patients developed secondary hypothyroidism (Nos. 1 and 13), one patient developed subclinical hyperthyroidism (No. 8), and one patient developed subclinical hypothyroidism (No. 11). Of the three patients with abnormal thyroid function tests prior to the 1st cycle of chemotherapy, all had euthyroid after the 5th cycle (Nos. 5, 10, and 14).

In terms of female gonadal function, there was no significant difference in LH, FSH, or estradiol before and after chemotherapy. In one female (No. 2), no secondary amenorrhea was reported after the 5th cycle of chemotherapy and no hypogonadism was documented based on FSH, LH, and estradiol levels.

**Figure 2:**
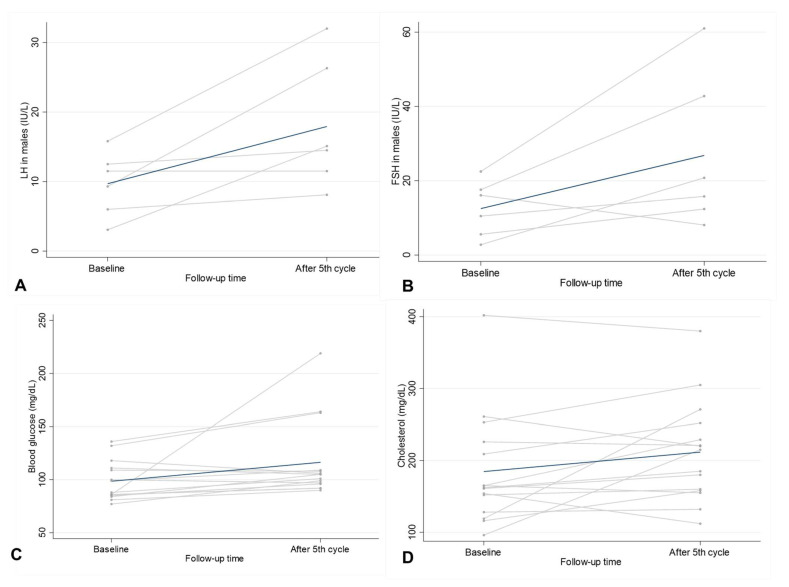
Trend of hormonal and metabolic changes at baseline and after 5th R-CHOP chemotherapy. (**A**) LH in males, (**B**) FSH in males, (**C**) blood glucose, and (**D**) cholesterol.

A comparison of male gonadal function before the 1st and after the 5th cycle of chemotherapy showed significantly higher LH levels after the 5th cycle (*p* = 0.040) and a trend toward significantly higher FSH levels; however, there was no significant difference in testosterone levels before and after the five cycles (Figure 2). Among the men with normal serum testosterone levels before the 1st cycle, two of five patients (Nos. 1 and 5) developed low testosterone in the hypogonadism range with non-suppressed FSH and LH levels after the 5th cycle of chemotherapy. There was also no significant difference in FSH or LH levels before and after chemotherapy in this group (*p* = 0.368 and *p* = 0.425 for FSH and LH, respectively). Of the man with low testosterone prior to the 1st cycle, an increase in testosterone level (No.10) after the 5th cycle was observed.

Serum 8 AM cortisol and peak serum cortisol after ACTH stimulation tests showed no significant difference in levels before and after chemotherapy. Three patients developed AI after the 5th cycle of chemotherapy (Nos. 1, 9, and 12). Their mean age was 62.7 ± 15.1 years, and their mean BMI was 24.5 ± 8.0 kg/m^2^. All patients with AI had good ECOG performance, bulky disease, and Ann Arbor stage > 2. Among those patients, one (No. 12) had hyponatremia and AI symptoms (lethargy and vomiting). One patient with AI also developed secondary hypothyroidism and male hypogonadism after the 5th cycle (No. 1). The best cut-off for serum cortisol at 8 AM to predict the occurrence of AI after the 5th cycle was <9.6 µg/dL with a sensitivity of 70.8% and specificity of 84.8%.

Summary data of hormonal functions after the 5th R-CHOP cycle as well as changes before the 1st and after the 5th R-CHOP cycle, are shown in Table 2 and Supplementary Table S2.

### 3.3. Metabolic Parameters before the 1st and after the 5th R-CHOP Chemotherapy Cycle

There was a trend toward significantly higher fasting blood glucose and serum cholesterol levels after the 5th cycle compared to before the 1st cycle of chemotherapy, as shown in Figure 2 (*p* = 0.061 and *p* = 0.072, respectively). Of the 11 patients without diabetes mellitus, the blood glucose of three patients changed from normoglycemic ranges to impaired fasting glucose ranges after the 5th cycle (Nos. 6, 7, and 13). Of the four patients with diabetes mellitus, all had a non-statistically significant increase in blood glucose levels after 5th R-CHOP chemotherapy (*p* = 0.13). No hypoglycemia events were documented after the 5th cycle of chemotherapy. Significantly lower serum creatinine after the 5th cycle compared to before the 1st cycle was also observed (*p* = 0.04) (Table 2 and Supplementary Table S2).

### 3.4. Nutritional Status and Anthropometric Measurements before the 1st and after the 5th R-CHOP Chemotherapy Cycle

Based on NAF scores, among the patients with normal to mild malnutrition status, 2 out of 13 patients developed a moderate degree of malnutrition after the 5th cycle of chemotherapy (Nos. 4 and 12). The patients with moderate malnutrition status at baseline were still in the same range after the 5th cycle (Nos. 1 and 11). A significantly higher prevalence of moderate malnutrition was observed after the 5th cycle than before the 1st cycle of chemotherapy (*p* = 0.01). For anthropometric data, the percentage of body water was significantly higher after the 5th cycle than before the 1st cycle (*p* = 0.02). Muscle mass and visceral fat levels revealed no significant difference before the 1st and after the 5th cycle of chemotherapy. Data are shown in Table 3.

## 4. Discussion

The R-CHOP regimen itself has been reported to cause multiple adverse systemic effects, including endocrine and metabolic abnormalities. This prospective cohort study demonstrated that hormonal dysfunctions, including male hypogonadism, secondary hypothyroidism, subclinical hypothyroidism, subclinical hyperthyroidism, and AI, can occur after the 5th cycle of the R-CHOP regimen for DLBCL. Other metabolic abnormalities involving increased glucose levels and deterioration in nutritional status can be observed in these patients as well.

The thyroid function abnormalities observed in the present study, secondary hypothyroidism, and subclinical hyperthyroidism, could result from suppression of TSH, FT3, and FT4 production resulting from high dose glucocorticoid therapy. One study reported that hypercortisolism could lower hypothalamic TRH expression and lead to TSH suppression [15]. Other studies have stated that glucocorticoid causes the suppression of TSH, T3 and T4 levels. These thyroid function alterations rose back to the normal range after glucocorticoid had been withdrawn [16,17], i.e., these effects were transient. Another cause of secondary hypothyroidism, which may not be associated with chemotherapy, is hypophysitis from pituitary infiltration of DLBCL [18]. This could explain the occurrence of both secondary hypothyroidism and AI in patient No. 1 in the present study. However, the evidence against that conclusion is that the stage of DLBCL in that patient was not advanced, and there were no hormonal dysfunctions before the 1st cycle of chemotherapy. We assume that after the 5th cycle, disease progression should either stabilize or improve, not progress. However, in the present study, pituitary MRI was not performed. Therefore, the cause of hormonal alterations in this patient could not be definitively determined. The elevated TSH level after the 5th cycle of chemotherapy could result from a hypothyroidism phase after thyroiditis. However, there have been no reports that any of the components in the R-CHOP regimen can induce thyroiditis. The R-CHOP regimen can, however, lead to complete resolution of autoimmune thyroiditis and can induce Graves’ hyperthyroidism, which was not observed in the present study [19,20]. Another possible explanation for high TSH level after the 5th chemotherapy cycle could be the recovery phase of NTI, in which elevated TSH can be observed [21]. In the future, follow-up thyroid function tests in these patients should be conducted to document this condition.

There were two patients with NTI prior to the 1st chemotherapy (Nos. 10 and 14). Their thyroid function tests had returned to the euthyroid state after the 5th cycle. A plausible explanation is that their clinical conditions improved after chemotherapy. The supportive evidence is that both patients’ BMI increased, and one patient had an improved malnutrition status based on NAF scores after the 5th chemotherapy. One patient with subclinical hyperthyroidism before chemotherapy also achieved a euthyroid state after the 5th cycle (No. 5). A possible explanation is that this patient may have had autoimmune hyperthyroidism and that the R-CHOP regimen may resolve that issue [19]. However, in the present study, markers of autoimmune diseases, e.g., TSH receptor antibodies or anti-TPO, were not retrieved.

In terms of gonadal function, only males showed gonadal dysfunction after the 5th cycle chemotherapy (Nos. 1 and 5). Primary hypogonadism with low testosterone and non-suppressed gonadotropin levels were observed. A high probability of gonadotoxicity resulting from cyclophosphamide and doxorubicin has been reported in multiple studies, while a low probability of gonadal damage has been reported with vincristine therapy [5,6,22]. The pathogenetic explanation was that cytotoxic chemotherapy could lead to direct toxic effects on Leydig cell function as well as spermatogenesis [23].

Regarding adrenal function, 3 out of the 15 patients (20%) had AI after the 5th cycle of chemotherapy [7]. Similar to the present study, one study reported transient AI in 3 out of 10 patients (30%) with DLBCL who received R-CHOP/CHOP chemotherapy. Their HPA axes were fully recovered at 3 to 5 weeks after completing a course of chemotherapy [10]. That study reported cut-off levels for basal cortisol of <8.7 µg/dL, which can indicate AI, comparable to our study results of 8 AM cortisol < 9.6 µg/dL. However, that study did not report other hormonal abnormalities as was carried out in the present study. The underlying pathophysiology is that long-term use of glucocorticoid can suppress the HPA axis, leading to tertiary AI by decreasing corticotropin-releasing hormone (CRH) synthesis and secretion, and the suppression of ACTH release by the anterior pituitary gland. As a result of the absence of ACTH, the adrenal cortex loses the ability to produce cortisol. Apart from glucocorticoids, cyclophosphamide has been reported to cause secondary AI in children with a hematopoietic stem cell transplant [24]. There have been no recent reports of other cytotoxic chemotherapies in the R-CHOP regimen that could induce primary AI from adrenal gland destruction. All patients who developed AI in our study had bulky disease with advanced stage DLBCL (Ann Arbor stages 3 and 4). Additionally, adrenal gland metastasis should be considered a possible differential diagnosis. The present study did not determine ACTH levels; thus, whether AI was a primary or secondary/tertiary could not be determined.

For glucose metabolism, increased glucose levels toward impaired fasting ranges were observed after the 5th cycle of chemotherapy. Glucocorticoids can stimulate gluconeogenesis from the liver, increase lipolysis from fat cells, and cause insulin resistance in peripheral tissue, including muscle [25]. Additionally, cyclophosphamide and doxorubicin have been reported to cause pancreatitis, which can lead to hyperglycemia [26]. However, no signs and symptoms of pancreatitis were reported in the present study.

The present study also observed significantly worsening nutritional status. A previous study of nutritional status in DLBCL patients found that malnutrition during R-CHOP chemotherapy was associated with worse survival outcomes and the occurrence of treatment-related toxicity [27]. In our study, among patients with moderate malnutrition at baseline, one developed secondary hypothyroidism, hypogonadism, and AI after the 5th cycle of chemotherapy (No. 1). One patient with worsening malnutrition status developed AI after the 5th cycle of chemotherapy (No. 12). A previous study stated that malnutrition might increase the risk of AI due to decreased excretion of 17-ketosteroids [28]. An increased percentage of body water was also observed in our study. Increased exercise or physical activity programs have been reported to be related to decreased body water mass in cancer patients [29]. It could be that the increased percentage of body water in our cohort may have been related to the sedentary lifestyle of the patients; however, data on physical activity was not collected in the present study.

This study had multiple strengths. First, this is the first study to demonstrate multiple hormonal and metabolic changes in DLBCL patients receiving R-CHOP chemotherapy. This data is of value to clinical practitioners taking care of lymphoma patients receiving R-CHOP chemotherapy. Clinical practitioners should be alert and have careful observation especially after the 5th cycle of R-CHOP chemotherapy, as some of the hormonal alterations, e.g., adrenal insufficiency, can be lethal. Second, nutritional status and anthropometric measurements were obtained during the study period. Third, ACTH stimulation tests were performed in all participants to diagnose AI.

There are also some limitations to this study. Initially, we anticipated a larger number of participants, but due to the COVID-19 situation, only a small number of patients were able to be recruited. However, based on sample size calculation, 15 patients with DLBCL were required to provide an adequate power of analysis. Another weakness is that the causes of AI after the 5th cycle of chemotherapy were not established. Similarly, whether the cause of secondary hypothyroidism was from R-CHOP or hypophysitis could not be definitely determined. After completing the chemotherapy regimen, long-term follow-up of multiple hormonal measurements was not performed as these identified changes can be transient. No control group was included in this study as, in our institution, all DLBCL patients had received chemotherapy. Therefore, whether the hormonal and metabolic changes were due to chemotherapy or the disease per se, could not be concluded. Finally, only one female of a reproductive age was included in our study, so female gonadal dysfunction could not be clearly evaluated.

## 5. Conclusions

In conclusion, multiple hormonal dysfunctions and metabolic abnormalities can occur in DLBCL patients after receiving the R-CHOP regimen. Clinical practitioners should be alert, especially after the 5th cycle of chemotherapy, to the occurrence of hormonal abnormalities, as some can be lethal.

## Figures and Tables

**Table 1:** Baseline characteristics and laboratory tests.

Characteristics	Value
Mean age, Mean ± SD (years)	60.7 ± 12.4
Sex	
Male, *n* (%)	6 (40)
Underlying disease, *n* (%)	8 (53.3)
DM, *n* (%)	4 (26.7)
HT, *n* (%)	6 (40)
DLP, *n* (%)	4 (26.7)
CKD, *n* (%)	1 (6.7)
Chronic hepatitis C, *n* (%)	1 (6.7)
Performance status, *n* (%)	
PS 0	10 (66.7)
PS 1	5 (33.3)
PS 2	-
Ann Arbor stage, *n* (%)	
1	1 (6.7)
2	4 (26.7)
3	4 (26.7)
4	6 (40)
B-symptom, *n* (%)	3 (20)
Bulky disease, *n* (%) #	12 (80)
Body weight, Mean ± SD (Kg)	54.9 ± 14.2
BMI, Mean ± SD (Kg/m^2^)	21.7 ± 4.4
Blood pressure (sitting position), Mean ± SD (mmHg)	122.8 ± 17.1
Blood pressure (supine position), Mean ± SD (mmHg)	119.6 ± 16.1
Adrenal insufficiency symptoms	
Vomiting, *n* (%)	2 (13.3)
Lethargy, *n* (%)	4 (26.7)
Orthostatic hypotension, *n* (%)	1 (6.7)
Female	
Menopause, *n* (%)	8 (88.9)
Amenorrhea in reproductive woman, *n* (%)	0

DM: diabetes mellitus, HT: hypertension, DLP: dyslipidemia, CKD: chronic kidney disease, PS: Performance status, BMI: body mass index, SD: standard deviation. # Tumor size ≥ 7.5 cm.

**Table 2:** Hormonal features and metabolic profiles before 1st cycle and after 5th cycle of R-CHOP.

Laboratory Tests	Baseline	After 5th Cycle	*p*-Value
Hb (Mean ± SD) (g/dL)	11.3 ± 2.3	10.6 ± 2.0	0.164
Cr (Mean ± SD) (mg/dL)	0.9 ± 0.5	0.8 ± 0.3	0.047
Na (Mean ± SD) (mEq/L)	138.1 ± 3.9	138.9 ± 3.9	0.515
K (Mean ± SD) (mEq/L)	4.0 ± 0.5	4.0 ± 0.4	0.914
HCO3 (Mean ± SD) (mEq/L)	25.3 ± 2.8	26.1 ± 2.4	0.235
LDH (Median, IQR) (U/L)	217 (185,248)	290 (237,233)	0.920
Albumin (Mean ± SD) (g/dL)	3.9 ± 0.5	3.9 ± 0.4	0.767
AST (Mean ± SD) (U/L)	27 ± 10.3	25.9 ± 6.3	0.621
Cholesterol (Median, IQR) (mg/dL)	162 (128,226)	215 (158,252)	0.072
Blood glucose (Median, IQR) (mg/dL)	88 (85,111)	105 (96,109)	0.061
FT4 (Mean ± SD) (ng/dL)	1.3 ± 0.3	1.1 ± 0.2	0.147
FT3 (Median, IQR) (pg/mL)	2.4 (2.2,2.6)	2.6 (2.3,2.9)	0.164
TSH (Mean ± SD) (μIU/mL)	1.8 ± 1.1	1.8 ± 1.5	0.973
FSH (Mean ± SD) (IU/L) Male Female Menopause Reproductive woman (*n* = 1)	12.5 ± 7.5 74.7 ± 25.2 9.5	26.8 ± 20.7 69.6 ± 18.0 3.1	0.082 0.497 -
LH (Mean ± SD) (IU/L) Male Female Menopause Reproductive age (*n* = 1)	9.7 ± 4.6 48.9 ± 16.9 14.2	17.9 ± 9.2 45.6 ± 9.8 1.7	0.040 0.401 -
Estradiol (Mean ± SD) (pg/mL) Menopause Reproductive age (*n* = 1)	11.2 ± 8.4 45.8	11.7 ± 9.5 63	0.832 -
Testosterone (Median, IQR) (ng/mL)	4.1 (3.2,5.3)	3.7 (2.7,6.1)	0.528
8.00 AM cortisol (Mean ± SD) (μg/dL)	12.4 ± 4.4	10.3 ± 5.2	0.179
Peak cortisol after ACTH stimulation test (Mean ± SD) (μg/dL)	20.3 ± 2.9	18.6 ± 6.4	0.383

Hb: hemoglobin level, Cr: creatinine, Na: sodium, K; potassium, SD: standard deviation, IQR: interquartile range.

**Table 3:** Nutritional status and anthropometric measurements before 1st cycle and after 5th cycle of R-CHOP.

Laboratory Testing	Baseline	After 5th Cycle	*p*-Value
NAF score, *n* (%)			
0–5 = normal-mild malnutrition	13 (86.7)	11 (73.3)	0.011
6–10 = moderate malnutrition	2 (13.3)	4 (26.7)	
≥11 = severe malnutrition	0	0	
Muscle mass (kg) (Median, IQR)	20.7 (16.1,28.2)	18.8 (17.6,27.8)	0.344
Body water (Mean ± SD) (%)	52.7 ± 4.2	55.4 ± 6.0	0.024
Visceral fat level (Median, IQR)	9 (8,12)	10 (10,12)	0.452

NAF: nutrition alert form, SD: standard deviation, IQR: interquartile range.

## Data Availability

The data will be provided upon a reasonable request.

## Supplementary Materials

The following supporting information can be downloaded at: https://www.mdpi.com/article/10.3390/medicina58060710/s1, Table S1: Summary of characteristics and laboratory tests prior to the 1st R-CHOP cycle in 15 patients; Table S2: Characteristics and laboratory tests after the 5th R-CHOP cycle in 15 patients.
