# Supplementary material for: Hormonal Changes after R-CHOP Chemotherapy in Patients with Diffuse Large B-Cell Lymphoma: A Prospective Study
Source: Medicina (Kaunas). 2022 May 26;58(6):710. doi: 10.3390/medicina58060710 (PMC9230452; doi:10.3390/medicina58060710)
Supplement: Supplementary file 1 [file medicina-58-00710-s001.zip › medicina-1714464-supplementary.pdf]

Table S1. Summary of characteristics and laboratory tests prior to the 1st R-CHOP cycle in 15 patients

Pt. No.	Sex	Age (years)	BMI (kg/m²)	UD	BP Sitting (mmHg)	PS	AAS	B-symptom	Bulky disease	AI symptoms	Sig. wt. loss	Amenorrhea	Male hypogonadism symptoms	Hb (g/dL)	Cr (mg/dL)	Na (mEq/L)	K (mEq/L)	FT_4 (ng/dL)	FT_3 (pg/mL)	TSH (µIU/mL)	FSH (IU/L)	LH (IU/L)	E (pg/mL)	T (ng/mL)	8.00 AM cortisol (µg/dL)	Peak cortisol (µg/dL)	BS (mg/dL)	NAF score
1	M	80	21.7	DM, HT, DLP, CKD	138/80	0	2	N	Y	-	N	-	N	12.7	1.1	139	4.2	1.55	2.24	1.29	17.6	9.3	0	17.6	12.7	24.5	86	9
2	F	34	17.3	-	140/90	0	1	N	N	-	N	N	-	12.3	0.6	143	3.5	1.33	2.83	3.18	9.5	14.2	45.8	-	8.05	18.5	85	5
3	F	56	23.5	-	118/70	0	2	N	N	-	N	MP	-	15.6	0.6	142	3.8	1.17	2.44	1.87	101.2	50.5	5.8	-	14.7	21.2	81	0
4	F	76	19.0	HT	148/70	1	4	N	Y	-	N	MP	-	12.6	1.2	137	4.7	1.21	2.42	1.01	66.6	62.8	5.3	-	7.4	21.9	111	1
5	M	79	20.9	DM, HT	133/59	1	4	Y	Y	Lethargy	N	-	N	8.5	2.3	137	4.2	1.72	2.55	0.25	22.5	15.8	-	3.18	13.6	20.9	132	4
6	F	53	17.4	-	120/70	0	3	N	Y	Lethargy	N	MP	-	11.9	0.6	128	4.8	1.33	2.36	3.75	95.4	54.9	5	-	16.5	26.6	99	2
7	M	68	19.6	-	96/59	0	4	N	Y	-	N	-	N	12.7	0.7	142	3.8	1.23	2.35	3.43	16.1	6	-	5.3	10.8	17.8	84	0
8	M	67	24.4	DM	129/72	0	2	N	N	-	N	-	N	8.9	0.8	138	3.8	0.78	2.49	2.04	10.5	12.5	-	4.5	9.5	17.2	77	3
9	M	52	33.5	-	135/85	0	3	N	Y	-	N	-	N	14.2	1.1	138	4.7	1.76	2.72	1.63	5.6	11.5	-	3.8	9.9	19.3	100	1
10	M	50	19.6	-	123/90	0	3	N	Y	-	N	-	N	8.8	0.6	136	3.2	1.02	1.05	1.02	2.8	3.07	-	2.1	9.34	19.1	86	0
11	F	61	22.2	DM, HT, DLP	125/69	0	3	N	Y	-	N	MP	-	11.2	1.5	139	4.5	1.59	2.32	3.26	81	61	6.7	-	16.3	19.1	136	6
12	F	56	17.7	HT	83/54	1	4	Y	Y	Vomiting, Lethargy, OH	N	MP	-	10.8	0.7	140	3.7	1.19	2.09	0.72	105	70	5	-	12.04	15.96	109	3
13	F	52	22.6	DLP	110/70	1	4	Y	Y	-	N	MP	-	8.8	0.5	134	4	1.06	2.87	1.16	40.9	19.6	24.8	-	23.6	22.8	88	1
14	F	66	18.3	-	100/56	1	4	N	Y	Vomiting, Lethargy	N	MP	-	7.8	1.0	136	3.6	1.27	1.46	0.71	42.4	37.1	22.9	-	14.6	21.9	118	4
15	F	60	28.0	HT, DLP	96/68	0	2	N	Y	-	N	MP	-	12.4	0.7	143	3.5	1.06	2.44	1.59	65	35	14	-	6.84	25.7	88	0

Pt. No., Patient number; F, Female; M, Male; BMI, Body mass index; UD, Underlying disease; DM, Diabetic mellitus; HT, Hypertension; DLP, dyslipidemia; CKD, chronic kidney disease; AI, Adrenal insufficiency; Sig. Wt. loss, Significant weight loss; BPsit, Blood pressure on sitting position; PS, Performance status; AAS, Ann Arbor stage; Y, Yes; N, No; OH, Orthostatic hypotension; MP, Menopause; A, Andropause; Hb, Hemoglobin; Cr, Creatinine; Na, Sodium; K, Potassium; FT4, Free thyroxine; FT3, Free 3,5,3'-triiodothyronine; TSH, Thyroid stimulating hormone; FSH, follicle stimulating hormone; LH, luteinizing hormone; E, Estradiol; T, Testosterone; BS, Blood sugar; NAF, Nutrition alert form

Table S2. Characteristics and laboratory tests after the 5th R-CHOP cycle in 15 patients

Pt, No.	BMI (kg/m^2)	AI symptoms	Sig wt loss	Amenorrhea	Male hypogonadism symptoms	Hb (g/dL)	Cr (mg/dL)	Na (mEq/L)	K (mEq/L)	FT$_4$ (ng/dL)	FT$_3$ (pg/mL)	TSH (µIU/mL)	FSH (IU/L)	LH (IU/L)	E (pg/mL)	T (ng/mL)	8.00 AM cortisol (µg/dL)	Peak cortisol (µg/dL)	BS (mg/dL)	NAF score
1	23.0	-	N	-	N	10.9	0.9	138	4	0.9	1.28	0.66	42.8	26.3	-	2.28	1.85	5.75	219	7
2	18.0	-	N	N	-	13	0.6	138	3.5	1.35	2.31	1.56	3.09	1.7	63	-	10.71	20.88	96	2
3	24.4	-	N	MP	-	14	0.6	143	3.8	0.95	2.65	2.75	85.5	44.2	5.7	-	18.4	26.8	90	1
4	17.1	Vomiting, lethargy	Y	MP	-	10.6	0.9	140	3.7	1.07	2.97	1.89	77.2	61.2	5.7	-	7.7	20.9	105	6
5	21.3	Lethargy	N	-	N	7.4	1.7	132	4.7	1.13	2.06	0.65	61	32	-	2.68	12.5	17.8	163	4
6	17.0	Lethargy	N	MP	-	9.7	0.7	133	4.5	0.99	2.75	2.57	88	43	5	-	8.09	17.3	109	2
7	18.4	-	N	-	N	13.7	0.8	141	3.7	1.29	2.42	2.35	8.1	8.1	-	6.54	9.06	17.7	105	4
8	26.8	-	N	-	N	10	0.7	142	4.2	1.08	3.68	0.008	15.8	14.5	-	3.49	6.02	15	99	3
9	33.1	-	N	-	N	11.8	0.9	140	4.2	1.18	3.89	1.54	12.4	11.5	-	3.9	5.7	14.2	97	1
10	22.6	-	N	-	N	11.5	0.7	143	4	1.21	2.84	1.01	20.8	15.1	-	6.1	14.6	21.5	92	1
11	20.5	-	N	MP	-	7	1.4	134	4.5	1.66	2.45	6.32	79	58	13.8	-	16.1	26.4	164	8
12	17.3	Vomiting, Lethargy	N	MP	-	9.2	0.7	134	3.4	1.05	2.27	1.42	60	49	5	-	2.35	6.02	108	6
13	23.0	-	N	MP	-	9.2	0.4	142	4.2	0.89	1.86	0.98	32	35	32	-	16.8	23.6	101	0
14	20.5	-	N	MP	-	9.7	0.68	142	4.3	1.21	2.79	0.61	64	39	8.54	-	10.4	20.2	106	1
15	24.8	Lethargy, OH	N	MP	-	10.8	0.7	142	3.5	1.42	3.11	2.75	71	35	18	-	14.9	25.7	92	5

Pt. No. Patient number; BMI, Body mass index; Y, Yes; N, No; OH, Orthostatic hypotension; MP, Menopause; A, Andropause; Hb, Hemoglobin; Cr, creatinine; Alb, albumin; Chol, cholesterol; FT4, free thyroxine; FT3, free 3,5,3'-triiodothyronine; TSH, thyroid stimulating hormone; FSH, follicle stimulating hormone; LH, luteinizing hormone; E, Estradiol; T, Testosterone; BS, Blood sugar; NAF, Nutrition alert form
